# Advances in management of primary myelofibrosis and polycythaemia vera: Implications in clinical practice

**DOI:** 10.1002/jha2.734

**Published:** 2023-06-07

**Authors:** Shanti Amé, Fiorenza Barraco, Jean‐Christophe Ianotto, Eric Jourdan, Jérôme Rey, Jean‐François Viallard, Mathieu Wémeau, Jean‐Jacques Kiladjian

**Affiliations:** ^1^ Department of Haematology Institut de Cancérologie Strasbourg Europe (ICANS) Strasbourg France; ^2^ Department of Haematology Lyon Sud Hospital Centre Pierre‐Bénite France; ^3^ Department of Clinical Haematology University Hospital of Brest Brest France; ^4^ Department of Clinical Haematology University Hospital of Nimes Nimes France; ^5^ Department of Haematology Institute Paoli‐Calmettes Marseille France; ^6^ Department of Internal Medicine Bordeaux University Hospital Bordeaux France; ^7^ Department of Haematology Hospital Centre of Roubaix Roubaix France; ^8^ Centre d'Investigations Cliniques Hôpital Saint‐Louis, Université Paris Cité Paris France

**Keywords:** diagnosis, myeloproliferative neoplasms, polycythaemia vera, primary myelofibrosis, prognosis, treatment

## Abstract

Primary myelofibrosis (PMF) and polycythaemia vera (PV) are rare *BCR‐ABL1*‐negative myeloproliferative neoplasms, associated with an increased risk of thrombosis, haemorrhagic complications and progression to fibrosis or leukaemia or fibrosis for PV. Both diseases are characterised by biological and clinical heterogeneity, leading to great variability in their management in routine clinical practice. In this review, we present an updated overview of the diagnosis, prognosis and treatment of PMF and PV, and we discuss how our multidisciplinary expert group based across France translates this evidence‐based knowledge into routine clinical practice.

## 1 INTRODUCTION

Primary myelofibrosis (PMF) and polycythaemia vera (PV) are *BCR‐ABL1*‐negative myeloproliferative neoplasms (MPNs) (**Table** **1**), which share mutations that constitutively activate the Janus kinase/signal transducer and activator of transcription (JAK/STAT) signalling pathway involved in haematopoiesis.[1, 2, 3] PMF is characterised by bone marrow (BM) megakaryocytic proliferation as well as reticulin and/or collagen fibrosis, whereas PV involves uncontrolled red blood cell (RBC) production.[4] Since both PMF and PV are heterogeneous disorders and given the growing body of molecular data on MPNs and consequently the expanding targeted therapies available, decisions regarding their diagnosis and treatment are often complicated.[5, 6] Accordingly, in this article, we present an updated overview of the diagnosis, prognosis and treatment of PMF and PV, and we discuss how our multidisciplinary expert group based across France translates current evidence into routine clinical practice. This paper was developed following several regional expert meetings, each led by one of the authors to discuss their real‐world experience in the management of PMF and PV. Regional meetings were followed by a national meeting during which the authors discussed and summarised the findings. The resulting manuscript was prepared and revised several times by all authors until they concurred on the final text and illustrations.

**TABLE 1:** 2022 World Health Organisation classification of myeloproliferative neoplasms [4].

Myeloproliferative neoplasms (MPNs)
Chronic myeloid leukaemia
Chronic neutrophilic leukaemia
Chronic eosinophilic leukaemia
Juvenile myelomonocytic leukaemia
Essential thrombocythaemia
Polycythaemia vera
Primary myelofibrosis
MPN, not otherwise specified

## 2 EPIDEMIOLOGY

PMF and PV are both rare disorders, with an incidence of 0.5–1.5 cases per 100,000 individuals for PMF and 0.7–2.6 cases per 100,000 for PV.[7] PMF and PV mainly occur in middle‐aged and older adults, with most patients being older than 60 years at initial diagnosis.[8]

## 3 DIAGNOSIS

PMF and PV can be asymptomatic at diagnosis and are sometimes discovered incidentally on a hemogram or after a routine clinical examination reveals a splenomegaly. Nevertheless, their diagnosis can be suspected based on several disease‐related symptoms. The hyperviscosity syndrome, characterised by headaches, dizziness, fatigue, visual or auditive disturbances, can affect PV patients. Aquagenic pruritus can also frequently alter the quality of life of these patients. In patients with PMF, common clinical manifestations include splenomegaly, bone pain, fatigue and constitutional symptoms (including weight loss, fever and sweating). Patients with PV or PMF both have an increased risk of thrombosis and haemorrhage, and such complications can also lead to diagnosis.[5, 7, 9–11]

In the current 2022 World Health Organisation (WHO) classification[4] and the International Consensus Classification (ICC) of myeloid neoplasms and acute leukaemias,[12] PV and PMF should be diagnosed by a combination of peripheral blood, BM morphologic and molecular features (**Table** **2**). In routine practice, diagnosis of PV can be presumptively made based on elevated haemoglobin and haematocrit. Isotopic measurement of the RBC mass can be useful to confirm a true polycythaemia in case of only a small increase in haematocrit, or in a particular context such as splanchnic vein thrombosis.[13, 14] It can also be helpful to distinguish 'masked PV' from essential thrombocythaemia (ET) in case of a haematocrit close to the upper limit (in 10–15% of all patients with PV), in the context of splenomegaly or haemodilution.[15] In PMF, the hemogram is usually quite characteristic, with the presence of immature granulocytes, erythroblasts and teardrop cells (dacrocytes). It can also show leucocytosis or leukopenia, as well as thrombocytosis or thrombocytopaenia. Anaemia is also frequent, with nearly 40% of PMF patients reporting haemoglobin levels < 10 g/dL at diagnosis and nearly one‐quarter already transfusion‐dependent.[10, 16, 17] As PMF progresses, it may potentially lead to severe cytopenia due to increase in BM fibrosis.[1]

**TABLE 2:** Diagnostic criteria for polycythaemia vera (PV) and primary myelofibrosis (PMF)[4, 12].

	PV	Prefibrotic PMF	Fibrotic PMF
**Major criteria**	**1**. Elevated haemoglobin (>16.5 g/dL in men or >16.0 g/dL in women) or haematocrit (> 49% in men or >48% in women) or increased red blood cell mass (>25% above mean normal predicted value)	**1**. BM biopsy showing megakaryocytic proliferation and atypia, with fibrosis grade 0 or 1, accompanied by increased age‐adjusted BM cellularity, granulocytic proliferation and often decreased erythropoiesis	**1**. BM biopsy showing megakaryocytic proliferation and atypia, accompanied by reticulin and/or collagen fibrosis grades 2 or 3
	**2**. Presence of JAK2V617F or *JAK2* exon 12 mutation	**2**. Presence of *JAK2*, *CALR* or *MPL* mutation or in the absence of these mutations, presence of another clonal marker (e.g. *ASXL1*, *EZH2*, *TET2*, *IDH1*/*IDH2*, *SF3B1*, *SRSF2*) or absence of reactive BM fibrosis	
	**3**. Bone marrow (BM) biopsy showing age‐adjusted hypercellularity with trilineage proliferation (panmyelosis), including prominent erythroid, granulocytic, and increase in pleomorphic, mature megakaryocytes without atypia	**3**. Not meeting the diagnostic criteria for other myeloid neoplasms	
**Minor criteria**	**1**. Subnormal serum erythropoietin level	Presence of ≥1 of the following, confirmed in 2 consecutive determinations:	
		**1**. Anaemia not attributed to a comorbid condition **2**. Leucocytosis ≥11×10[9]/L **3**. Palpable splenomegaly **4**. Lactate dehydrogenase (LDH) increased to above upper normal limit of institutional reference range	**1**. Anaemia not attributed to a comorbid condition **2**. Leucocytosis ≥11×10[9]/L **3**. Palpable splenomegaly **4**. LDH increased to above upper normal limit of institutional reference range **5**. Leukoerythroblastosis
**Requirements for diagnosis**	All three major criteria or first two major criteria + the minor criterion^a^	All three major criteria, and ≥1 minor criterion	All three major criteria, and ≥1 minor criterion

^a^ A BM biopsy may not be required in patients with sustained absolute erythrocytosis (haemoglobin concentrations > 18.5 g/dL in men or > 16.5 g/dL in women and haematocrit values > 55.5% in men or > 49.5% in women) and the presence of a JAK2V617F or *JAK2* exon 12 mutation.

The constitutive activation of the JAK/STAT pathway is a pathogenetic hallmark of all MPNs, with three well‐characterised driver mutations in *JAK2*, *CALR* and *MPL* genes.[3, 9] JAK2V617F, a valine‐to‐phenylalanine substitution at amino acid position 617 (V617F) in exon 14 of *JAK2*, is the most prevalent mutation in MPNs, present in > 95% of patients with PV and in approximately 60% of patients with PMF.[3, 18] *JAK2* exon 12 mutations are also observed in up to 3% of patients with PV. *CALR* and *MPL* mutations are present in up to 25% and 8% of patients with PMF, respectively.[3, 18]

### 3.1 Our group's practical applications

- Our group starts the diagnostic workup of suspected PMF or PV with a careful clinical examination of signs and symptoms associated with these pathologies such as splenomegaly.
- After clinical examination, we perform the following laboratory investigations:
- ○Complete blood count to detect elevated haemoglobin and haematocrit in patients with PV and anaemia in patients with PMF.
- ○Peripheral blood smear to detect immature granulocytes, erythroblasts and teardrop cells (dacrocytes) in patients with PMF.
- ○Serum lactate dehydrogenase, which is increased in both PMF and PV.
- ○Serum iron‐level measurement, as iron deficiency is a known feature of PV occurring because of accelerated erythropoiesis, and which can mask PV in patients with chronic bleeding.
- Abdominal ultrasound can be used to document and confirm splenomegaly.
- We perform *JAK2* molecular testing early on in the diagnostic workup of PV and PMF.
- In clinical practice, BM biopsy is not always performed to diagnose PV. In this perspective, our group reserves BM biopsies for specific situations such as:
- ○Masked PV.
- ○*JAK2*‐negative disease.
- ○In young patients.
- ○If there are atypical features such as marked splenomegaly or a history of splanchnic vein thrombosis.
- We invariably perform a BM biopsy to confirm PMF. However, the benefit/risk balance of BM biopsy should be discussed in elderly patients, those who have severe thrombocytopaenia, or on anticoagulation, if all other PMF diagnostic criteria are fulfilled. When the biopsy read is challenging, a second opinion is sought through a national group of experts specialised in BM histopathology.
- Our group performs cytogenetic analyses on BM aspirates, or in peripheral blood when BM cannot be obtained in PMF.
- Next‐generation sequencing (NGS) covering the whole *JAK2* gene is only useful in exceptional PV cases with atypical mutations outside exons 12 and 14.
- In suspected PMF cases, a targeted NGS panel providing detection of atypical mutations in *JAK2*, *CALR* and *MPL* genes should also be performed. Our group only requests a more extended NGS panel analysis to:
- ○Search for a clonality marker in patients with triple‐negative PMF.
- ○Assess high‐molecular risk mutations that confer poorer prognosis (e.g. *TP53*, *ASXL1*, *SRSF2*, *EZH2*, *IDH1/2*).
- ○Complement a doubtful BM biopsy.
- ○Guide therapeutic decision‐making.

## 4 PROGNOSIS AND RISK STRATIFICATION

Among the MPNs, PMF has the least favourable prognosis. A retrospective cohort study of 3023 patients reported a median overall survival (OS) of 18 years for ET (N = 1076), 15 years for PV (N = 665), and 4.4 years for PMF (N = 1282).[19]

Several risk assessment scoring systems have been developed taking into account the clinical, morphologic, cytologic, cytogenetic and/or molecular aspects of PMF (**Table** **3**). There is no consensus regarding the optimal prognostic tool for PMF, and these risk models have never been prospectively validated.[20] The choice of the risk stratification model may vary for each patient, according to available information.[6, 21] However, compared to the International Prognostic Scoring System (IPSS)[22] applied at diagnosis only, all other PMF risk models can estimate survival from any point in the disease course. Given the limited availability of molecular findings in routine clinical practice, dynamic IPSS (DIPSS)[23] and DIPSS‐plus[24] remain the most commonly used models for risk stratification and prognostication of PMF, as they are based on easily assessable clinical characteristics and blood counts.[6, 21] However, because of the prognostic relevance of the mutational profile in PMF,[25] we favour the use of mutation‐enhanced scores for transplantation‐age patients (MIPSS70 and MIPSS70‐plus).[26] In addition, the Myelofibrosis Secondary to PV and ET‐Prognostic Model (MYSEC‐PM) is a prognostic risk score specifically developed for patients with secondary myelofibrosis (SMF).[27] Scoring systems are overall valuable tools that are often used by multidisciplinary tumour boards (MTBs) to tailor management and therapeutic decisions of patients with PMF/SMF, especially regarding allogeneic haematopoietic stem cell transplantation (AHSCT).

**TABLE 3:** International prognostic scoring systems for primary myelofibrosis.

System		IPSS	DIPSS	DIPSS‐plus	MIPSS70	MIPSS70‐plus version 2.0	GIPSS
**Applicability**		At diagnosis	At any time				
**Online calculator**		No			http://www.mipss70score.it/		No
**Features**		Clinical		Clinical & molecular			Molecular
**Items (points)**	Age	>65 years (1)	>65 years (1)	>65 years (1)	–	–	–
	Hb	<10 g/dL (1)	<10 g/dL (2)	<10 g/dL (2)	<10 g/dL (1)	Moderate^a^ (1) Severe^b^ (2)	–
	WBC	>25 × 10[9]/L (1)	>25 × 10[9]/L (1)	>25 × 10[9]/L (1)	>25 × 10[9]/L (2)	–	–
	Peripheral blood blasts	≥1% (1)	≥1% (1)	≥1% (1)	≥2% (1)	≥2% (1)	–
	Constitutional symptoms	Yes (1)	Yes (1)	Yes (1)	Yes (1)	Yes (2)	–
	Platelets	–	–	<100 × 10[9]/L (1)	<100 × 10[9]/L (2)	–	–
	Red cell transfusion dependence	–	–	Yes (1)	–	–	–
	BM fibrosis	–	–	–	Grade ≥2 (1)	–	–
	Karyotype	–	–	Unfavourable^c^ (1)	–	Unfavourable^d^ (3) VHR^e^ (4)	Unfavourable^d^ (1) VHR^e^ (2)
	Mutations	–	–	–	No *CALR* type 1/like mutation (1) One HMR^f^ mutation (1) ≥2 HMR^f^ mutations (2)	No *CALR* type 1/like mutation (2) One HMR^f^ mutation (2) ≥2 HMR^f^ mutations (3)	No *CALR* type 1/like mutation (1) *ASXL1* (1) *SRSF2* (1) *U2AF1Q157* (1)
Risk groups (points): median OS, years		Low (0): 11.3 Int‐1 (1): 7.9 Int‐2 (2): 4 High (≥3): 2.3	Low (0): NR Int‐1 (1−2): 14.2 Int‐2 (3−4): 4 High (≥5): 1.5	Low (0): 15.4 Int‐1 (1): 6.5 Int‐2 (2−3): 2.9 High (≥4): 1.3	Low (0−1): NR Int (2−4): 6.3 High (≥5): 3.1	Very low (0): NR Low (1−2): 16.4 Int (3−4): 7.7 High (5−8): 4.1 Very high (≥9): 1.8	Low (0): 26.4 Int‐1 (1): 8 Int‐2 (2): 4.2 High (≥3): 2

^a^ Moderate anaemia: Haemoglobin (Hb) 8 to 9.9 g/dL.

^b^ Severe anaemia: Hb < 8 g/dL.

^c^ Complex karyotype or a single or two abnormalities, including + 8, −7/7q‐, i(17q), −5/5q‐, 12p‐, inv(3), or 11q23 rearrangement.

^d^ Any karyotype other than very high‐risk (VHR) karyotype, normal karyotype, or sole abnormalities of 20q‐, 13q‐, 19, chromosome 1 translocation/duplication, ‐Y, or sex chromosome abnormality other than ‐Y. .

^e^ Single/multiple abnormalities of −7, i(17q), inv(3)/3q21, 12p‐/12p11.2, 11q‐/11q23, or other autosomal trisomies not including +8/ +9 (e.g. +21, +19).

^f^ In MIPSS70, high‐molecular risk (HMR) mutations include *ASXL1*, *EZH2*, *SRSF2*, and *IDH1/2*; MIPSS70‐plus version 2.0 also includes mutated *U2AF1*.

Abbreviations: BM, bone marrow; DIPSS, dynamic IPSS; GIPSS, Genetically Inspired Prognostic Scoring System; Int, intermediate; IPSS, International Prognostic Scoring System; MIPSS70, Mutation‐Enhanced International Prognostic Score System for Transplantation‐Age Patients; NR, not reached, OS, overall survival; WBC, white blood cell.

In patients with PV, age and thrombotic history are the two major risk factors used for risk stratification in clinical practice. Patients aged > 60 years and/or having prior PV‐associated arterial or venous thrombosis are considered at “high risk” of thrombosis, whereas those ≤60 years with no PV‐associated thrombotic history are categorised as “low risk.”[5, 11, 28] However, it is important to also take into account cardiovascular risk factors (smoking, diabetes mellitus, arterial hypertension, hypercholesterolaemia), the presence of leucocytosis and the symptomatic burden associated with PV to guide risk stratification and subsequent therapeutic management.[11, 28, 29]

### 4.1 Our group's practical applications

- As a risk assessment scoring system for PMF, our group favours the use of MIPSS70 and MIPSS70‐plus incorporating both clinical and molecular features.
- In our practice, we encourage the submission of the medical file of every patient with newly diagnosed PMF/SMF or PV to MTBs. This serves to confirm diagnosis and obtain a multidisciplinary therapeutic decision that optimally benefits the patient. MTBs also provide an important forum to enhance patients’ therapeutic options including participation in clinical trials. For this reason, we rely on the presence, during MTB sessions, of a variety of specialists and healthcare professionals including biologists, pathologists and clinical research teams. There are however few PMF‐ and PV‐dedicated MTBs in France, with some centres relying on the MTB of another tertiary institution.

## 5 TREATMENT OF PMF

### 5.1 Allogeneic haematopoietic stem cell transplantation (AHSCT)

Currently, AHSCT is the only treatment modality in PMF that offers a potential cure.[9] According to consensus‐based recommendations by the European LeukemiaNet (ELN) and the European Blood and Marrow Transplantation (EBMT) Group, when using the DIPSS or DIPSS‐plus risk classification, all patients aged < 70 years with no major comorbidities and intermediate‐2 or high‐risk disease should be considered potential candidates for AHSCT.[30] For asymptomatic patients with DIPSS/DIPSS‐plus low or intermediate‐1 risk, a watch‐and‐wait approach is preferred because long‐term survival can be expected.[6] Our group considers patients with DIPSS/DIPSS‐plus intermediate‐1 risk PMF aged < 65 years as candidates for AHSCT if they have either refractory and transfusion‐dependent anaemia, a percentage of blasts in peripheral blood > 5%, or an unfavourable karyotype. In addition, in a recent analysis of the molecular landscape of 479 patients with PMF and SMF, mutations of *TP53*, *EZH2*, *CBL*, *U2AF1*, *SRSF2*, *IDH1*, *IDH2*, *NRAS* or *KRAS* were adverse prognostic factors in myelofibrosis, whereas *ASXL1* isolated mutations had no prognostic impact.[31] Hence, if a suitable donor is available, we also consider AHSCT in intermediate‐1 risk PMF patients with these adverse mutations.[30, 32]

**FIGURE 1:**
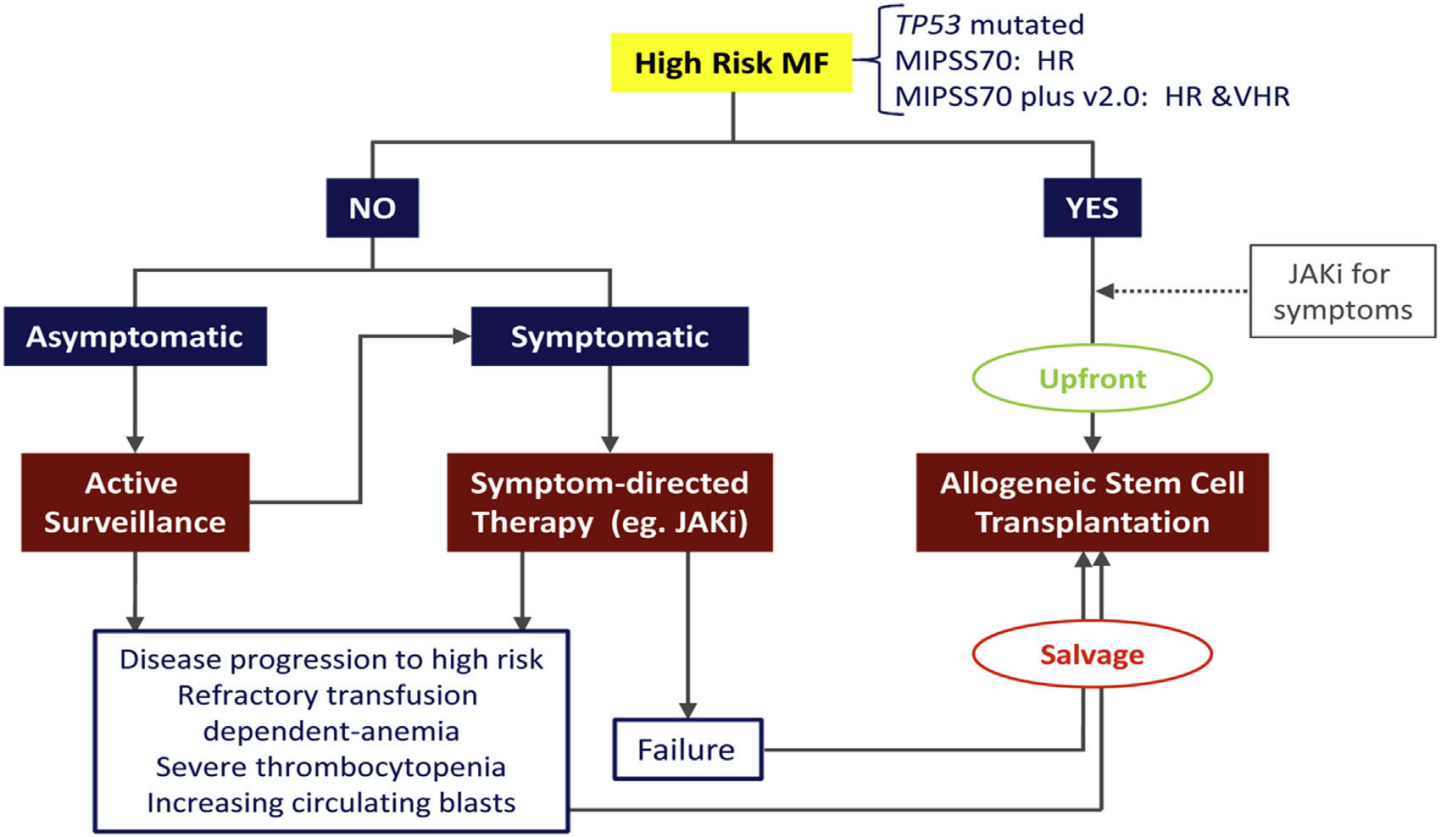
Treatment algorithm for patients with primary myelofibrosis (PMF). HR, high‐risk; JAKi, Janus kinase inhibitors; MIPSS70, Mutation‐Enhanced International Prognostic Score System for Transplantation‐Age Patients; VHR, very high risk.

Based on risk stratification according to MIPSS70‐plus version 2.0, AHSCT is the preferred treatment for very high‐ and high‐risk disease (**Figure** **1**). Observation alone is advised for low‐ and very low‐risk disease, whereas patients with intermediate‐risk disease are best served by receiving symptom‐directed conventional therapy or participating in clinical trials.[33]

AHSCT in PMF is associated with up to a 50% rate of transplant‐related mortality or severe morbidity (e.g. graft‐versus‐host disease (GVHD)).[34] The decision of performing AHSCT in patients with PMF is also complicated by the lack of consensus regarding donor type, the optimal source of the stem cell graft, the choice of conditioning regimen and the optimal timing of AHSCT. It is worthy to note that no randomised controlled trials (RCTs) have compared AHSCT with non‐transplant treatment strategies, and recommendations guiding transplant decisions are largely based on retrospective analyses and expert opinion.[20]

The timing of AHSCT is the most important question in PMF management.[2] Delaying AHSCT may lead to a worse outcome due to advanced‐stage disease, increasing age, worsening performance status or leukemic transformation. Conversely, if performed too early, AHSCT may significantly compromise quality of life due to treatment‐related morbidity. Nevertheless, it is best to not delay AHSCT to when disease progresses to avoid non‐engraftment and an increased risk of non‐relapse mortality (NRM).[35] Moreover, for patients progressing to the accelerated or blast phase of myelofibrosis, prognosis is poorer, as the probability of achieving long‐term disease control after transplant is severely reduced. Consequently, it is best to proceed to AHSCT as soon as possible.[35]

Peripheral blood remains the predominant and most appropriate stem cell source for AHSCT recipients with PMF.[20, 30, 32, 36] Regarding donor selection, as for other diseases, a human leukocyte antigen‐matched sibling donor followed by a matched unrelated donor are the preferred donor types, as they are associated with lower mortality and acute GVHD risk compared to other donor types.[20, 32, 37, 38] However, there is increasing evidence about the use of alternative donors (i.e. haploidentical and umbilical cord blood), which provide an attractive alternative source of stem cells due to widespread availability and ease of stem cell procurement.[30, 32, 37, 39]

Relapse is a major cause of treatment failure for patients with PMF after AHSCT, with an incidence up to 43%.[30] Detection of post‐transplant relapse may be challenging in clinical practice, as BM fibrosis may persist for months after AHSCT, and molecular monitoring is not yet standardised.[37] For patients with evidence of minimal residual disease or clinical relapse, discontinuation of immune‐suppressive drugs, donor lymphocyte infusions or a second AHSCT are treatment strategies of choice.[30, 32]

### 5.2 Our group's practical applications

- Although our group follows international guidelines regarding AHSCT in PMF,[30] we rely on a personalised, rather than a standardised approach, to evaluate candidates for AHSCT, taking into account disease‐, patient‐ and transplant‐related risk factors including older age, low performance status, high comorbidity burden and presence of portal hypertension.
- We carefully follow‐up transplant‐ineligible patients and re‐evaluate AHSCT indication in case of disease progression.
- AHSCT may be performed after leukemic transformation in selected patients.

### 5.3 Treatment of PMF‐associated splenomegaly

More than 50% of patients with myelofibrosis will develop splenomegaly during the course of their disease.[20] Splenomegaly may negatively affect AHSCT outcomes, as it may be associated with increased engraftment failure and mortality.[20, 32, 37] The mainstay of splenomegaly treatment in PMF is the use of oral JAK inhibitors, which are able to suppress inflammatory cytokines and inhibit JAK2‐mediated haematopoietic stem cell migration.[2, 33, 40] Ruxolitinib is the first JAK inhibitor approved by the United States Food and Drug Administration (FDA) in 2011 for the treatment of patients with intermediate‐ or high‐risk myelofibrosis and by the European Medicines Agency (EMA) in 2012 for the treatment of splenomegaly and/or other symptoms in patients with myelofibrosis, irrespective of mutational status.[32] Approval of ruxolitinib was based on the results of two phase III RCTs, COMFORT‐I and COMFORT‐II, in which ruxolitinib therapy for 24 weeks significantly reduced spleen volume and improved myelofibrosis‐related symptoms and quality of life, compared with placebo or best available therapy in patients with intermediate‐2 or high‐risk myelofibrosis and a platelet count ≥100 × 10^9^/L.[41, 42] In a pooled analysis of 5‐year data from the COMFORT trials, the risk of death was significantly reduced by 30% among patients randomised to ruxolitinib compared to the control group (p = 0.0065).[43] Consistent with the survival benefit of ruxolitinib in the RCT setting, a recent study, which prospectively collected real‐world data from 1010 European patients diagnosed with myelofibrosis from 2001 through 2012, reported improved median OS with ruxolitinib compared to other symptom‐directed therapies such as hydroxyurea (7.7 vs. 3.4 years, respectively; p = 0.002).[44]

While the clinical benefits of ruxolitinib are undeniable, its use has some limitations. Given the essential function of JAK2 in mediating signals from erythropoietin and thrombopoietin, dose‐dependent anaemia and thrombocytopaenia represent important practical challenges of ruxolitinib therapy.[45, 46] Ruxolitinib is particularly difficult to use in patients with severe thrombocytopaenia (platelets < 50×10^9^/L).[46] The starting dose of ruxolitinib in PMF is dependent on the patient's baseline platelet counts.[47] Ruxolitinib use is also limited by the fact that a large proportion of ruxolitinib‐treated patients will eventually develop clinical resistance or intolerance.[40] The prognosis for patients who discontinue ruxolitinib is dismal, with a reported median OS of only 13–14 months.[40, 46] The use of alternative JAK inhibitors (e.g. fedratinib, momelotinib and pacritinib) should be considered when ruxolitinib treatment fails.[35] Fedratinib has been approved by both the EMA and the FDA for the treatment of disease‐related splenomegaly or symptoms in adults with PMF who are JAK inhibitor‐naïve or previously treated with ruxolitinib.[48]

Spleen size may significantly impact engraftment and graft function, and constitutional symptoms are a risk factor for mortality. Pre‐transplant JAK inhibitor therapy with ruxolitinib is thus recommended by the ELN/EBMT in patients with symptomatic splenomegaly and/or constitutional symptoms to decrease spleen size and improve constitutional symptoms in order to reduce therapy‐related complications after AHSCT.[30] Ruxolitinib should be initiated ≥2 months before AHSCT and should be titrated to the maximum tolerated dose.[30] In 2016, Shanavas and colleagues[49] retrospectively analysed outcomes of 100 patients with myelofibrosis who underwent AHSCT with prior exposure to JAK inhibitors. Around one‐quarter of patients achieved clinical improvement while on JAK inhibitors, with a 2‐year OS rate significantly higher as compared to non‐responsive or progressive patients (91% versus 55%; p = 0.01).[49] More recently, in the JAK ALLO prospective phase II study of 64 patients with myelofibrosis who received ruxolitinib 15 mg twice daily for ≥3 months, the probability of undergoing AHSCT after ruxolitinib initiation was excellent (92%), with OS rates of 68% and 55% at 12 and 24 months, respectively.[38] In another recent EBMT registry‐based retrospective study of 551 patients with myelofibrosis, 277 (50.3%) of whom received ruxolitinib treatment prior to AHSCT, ruxolitinib pre‐treated patients with ongoing spleen response at transplant had a significantly lower risk of relapse (8.1% versus 19.1%; p = 0.04) and a better 2‐year event‐free survival (68.9% vs. 53.7%; p = 0.02) compared to patients without ruxolitinib pre‐treatment.[50]

Although these data appear to support pre‐transplant use of ruxolitinib, caution is advised. In all three aforementioned trials,[38, 49, 50] pre‐AHSCT ruxolitinib therapy was not beneficial for reducing the incidence of acute or chronic GVHD. Concerns about the safety of ruxolitinib in the pre‐AHSCT setting have also been raised. For instance, the JAK ALLO trial reported unusual serious adverse events of ruxolitinib, such as tumour lysis syndrome and cardiogenic shock.[38] Ruxolitinib may also be associated with a cytokine rebound syndrome if rapidly withdrawn.[50] According to ELN/EBMT recommendations, weaning of ruxolitinib starting 5–7 days prior to conditioning should be implemented in an attempt to avoid a cytokine rebound, with ruxolitinib discontinued the day before conditioning.[30] The role of ruxolitinib post‐AHSCT for response maintenance remains unclear, with very limited information to guide clinicians. However, ruxolitinib may be considered in patients who relapse after AHSCT to decrease the symptom burden.[32]

In patients who are refractory, intolerant to, or ineligible for JAK inhibitors, and who have a massive and symptomatic splenomegaly, splenectomy represents a valuable therapeutic strategy.[33, 40] Indications for splenectomy in myelofibrosis include splenic abdominal pain and discomfort, severe thrombocytopaenia and frequent RBC transfusions.[33] However, since splenectomy leads to perioperative complications (i.e. bleeding, infections, thrombocytosis) in approximately one‐third of patients with myelofibrosis and a perioperative mortality rate of up to 10%, the procedure should only be considered for carefully selected patients.[35, 51] In the post‐splenectomy setting, it is important to reinstitute JAK inhibitor therapy, if possible, to prevent accelerated hepatomegaly from extramedullary haematopoiesis in the liver.[40]

The role of splenectomy before AHSCT remains controversial, as data on splenectomy before AHSCT are inconsistent.[20, 32] In a recent EBMT registry‐based retrospective study of 1195 patients with myelofibrosis transplanted between 2000 and 2017, splenectomy was documented in 202 patients (16.9%) and was associated with decreased NRM but increased risk of relapse, with no impact on OS.[52] However, a survival benefit for splenectomy was shown in patients with marked splenomegaly, defined as a palpable spleen length ≥15 cm.[52] These results were also supported by a comprehensive retrospective study using the French BM transplantation registry (RFGM), which showed that pretransplant splenectomy did not preclude AHSCT in patients with PMF.[53] Based on available data, it is accepted that clinical decisions regarding pre‐AHSCT splenectomy should be made on an individualised basis.

Splenic irradiation is a well‐recognised alternative to splenectomy in patients with PMF who have symptomatic splenomegaly and are ineligible for surgical procedures.[54] Although radiation dosages vary widely, the most commonly administered dose for splenomegaly is reportedly 10 Gy in 10 fractions over 2 weeks.[55] Splenic irradiation is thought to have a role in reducing the number of neoplastic cells in the spleen, leading to improvements in both splenic size and discomfort. Unfortunately, the benefit of splenic irradiation is usually short‐lived, and severe pancytopenia is frequently observed if blood counts are not cautiously monitored (at least twice weekly) during irradiation.[33, 35] Data on the value of pre‐AHSCT splenic irradiation are scarce and inconclusive.[33]

### 5.4 Our group's practical applications

- We use ruxolitinib for the first‐line treatment of splenomegaly in patients with myelofibrosis.
- In our practice, we aim to find the optimal balance between efficacy and ruxolitinib‐related toxicities such as thrombocytopaenia and anaemia by performing a complete blood count at least every 2 weeks after ruxolitinib initiation and until ruxolitinib doses are stabilised.
- In case of clinical resistance or intolerance to ruxolitinib, we recommend fedratinib for reducing splenomegaly.
- In transplant‐eligible patients with symptomatic splenomegaly, we initiate pre‐transplant ruxolitinib therapy ≥2 months before AHSCT.
- In patients who are refractory, intolerant to, or ineligible for JAK inhibitors, and who have a massive and symptomatic splenomegaly, we perform splenectomy. As asplenism increases the risk of life‐threatening infections from encapsulated organisms, we always vaccinate patients undergoing splenectomy against *Streptococcus pneumoniae*, *Haemophilus influenzae*, and *Neisseria meningitidis*.
- In patients who have symptomatic splenomegaly and are ineligible for surgical procedures, we opt for splenic irradiation instead of splenectomy. In our practice, we usually monitor the blood count every 2 days in patients treated with splenic irradiation, and we hold irradiation if severe neutropenia (< 0.5×10^9^/L) or thrombocytopaenia (< 20×10^9^/L) is detected.

### 5.5 Treatment of PMF‐associated anaemia

Anaemia in PMF is generally multifactorial, and patients should be evaluated for bleeding, haemolysis, nutritional deficiencies (e.g. iron, vitamin B12, folate) and other remediable causes.[54] Available (off‐label) drugs for the management of anaemia in PMF include erythropoiesis‐stimulating agents (ESAs) such as epoetin and darbepoetin, corticosteroids (e.g. prednisone) and androgens (e.g. danazol).[35] Unfortunately, their efficacy is often limited and short‐lived.

Nearly all patients with PMF‐associated anaemia will eventually become transfusion‐dependent, requiring chronic RBC transfusions that may improve symptoms and quality of life.[54, 56] Complications of chronic RBC transfusions, however, include iron overload, cardiac insufficiency and alloimmunisation.[56] In patients with transfusion‐dependent anaemia and iron overload, iron chelation therapy is the main approach to prevent iron‐induced organ damage.[35, 54]

Splenectomy may exceptionally be indicated for palliative control of persistent anaemia.[54] It improves anaemia and thrombocytopaenia by allowing for sequestered blood cells to enter the peripheral circulation.[40] However, splenectomy is associated with significant morbidity and mortality, with the most common complications being bleeding, infection and thrombosis.[56, 57]

There are also several promising agents for improving PMF‐associated anaemia under prospective clinical evaluation. These include activin receptor ligand traps such as luspatercept, JAK inhibitors such as momelotinib and pacritinib, activin receptor‐like kinase 2 (ALK2) inhibitors like INCB000928 and the bromodomain and extraterminal domain protein (BET) inhibitor pelabresib.[35, 56, 58–60]

### 5.6 Our group's practical applications

- In patients with PMF‐associated anaemia, we propose ESAs in the context of erythropoietin levels < 125 U/L. However, we avoid ESAs in patients with marked splenomegaly, as they can activate the JAK pathway, potentially resulting in increased spleen size.
- We prefer corticosteroids or androgens for PMF‐associated anaemia in patients with marked splenomegaly and in the context of high serum erythropoietin levels.
- In transfusion‐dependent patients with PMF, chronic RBC transfusions increase the risk of iron overload. In our clinical practice, all patients with PMF who received > 20 RBC units and/or have a serum ferritin > 1000 ng/mL should be candidates for iron chelation therapy to reduce iron overload development.

## 6 TREATMENT OF PV

**FIGURE 2:**
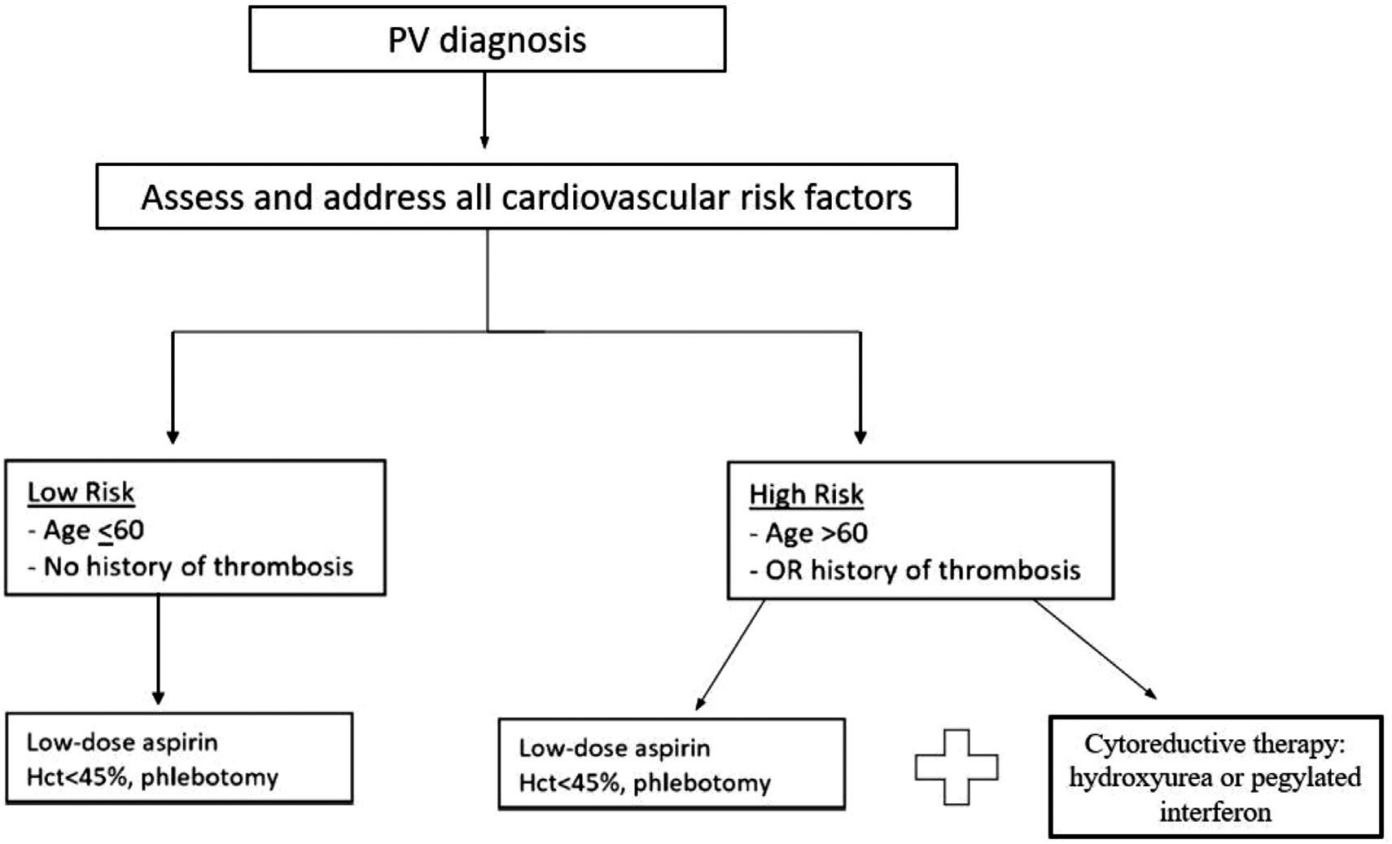
Risk‐stratified treatment algorithm of polycythaemia vera (PV). Hct, haematocrit.

Management of PV is primarily guided by the risk for thromboembolic events (**Figure** **2**), but also the chronicity of the disease and the long‐term risk of haematological transformation to SMF or acute leukaemia.[61] Low‐risk patients (aged ≤60 years with no history of thrombosis) should be treated with low‐dose aspirin (75–100 mg/day) and phlebotomy.[11] Phlebotomy should begin as soon as possible after the diagnosis of PV. In the induction phase, the phlebotomy regimen should consider a person's weight and should remove 300–450 mL of blood weekly or twice weekly until target haematocrit < 45% is achieved.[28] During the maintenance phase, phlebotomy intervals should be determined by measuring haematocrit levels monthly in the first 6 months and then every 1–2 months thereafter.[62] High‐risk patients (aged > 60 years and/or history of thrombosis) should receive, in addition to phlebotomy and low‐dose aspirin, cytoreductive therapy with hydroxyurea or pegylated interferon alpha, with the latter recommended in patients aged < 60 years and in women who desire pregnancy.[18, 63–66] Recently, the ELN proposed that cytoreductive therapy with interferon alpha should be considered in specific clinical subgroups of low‐risk patients with PV, especially in cases of intolerance to phlebotomy, progressive splenomegaly (increase by > 5 cm in the past year), persistent leucocytosis or thrombocytosis, or high symptom burden (e.g. severe pruritus).[29] Both low‐risk and high‐risk patients should be managed aggressively for cardiovascular risk factors, such as hypertension, hypercholesterolaemia, diabetes mellitus and smoking, as they also contribute to thrombotic risk in PV.[11, 28]

Based on its efficacy in controlling blood counts and preventing thrombosis, ease of administration, lower cost, wider availability and favourable toxicity profile, hydroxyurea, administered orally at a starting dose of 500 mg twice daily, is the most frequently used first‐line cytoreductive drug in PV.[11, 63, 64, 66] However, hydroxyurea is contraindicated in pregnancy, and is hence recommended to be stopped 3 months prior to intended conception.[11] By contrast, pegylated interferon is generally considered to be safe in pregnancy, and is therefore the preferred cytoreductive drug for young women of reproductive age.[66, 67] Although hydroxyurea is a manageable and well‐tolerated drug, during the course of disease, a quarter of patients with PV become resistant (12%) or intolerant (13%) to hydroxyurea.[68] Resistance to hydroxyurea therapy is an important adverse prognostic factor, associated with a sixfold increased risk of death and a sevenfold increased risk of progression to myelofibrosis or transformation to acute leukaemia.[68]

Second‐line cytoreductive therapy for PV includes pegylated interferon as a substitute for hydroxyurea and hydroxyurea for pegylated interferon.[11, 28, 66] Moreover, ruxolitinib was approved in the United States in 2014 and in Europe in 2015 for the treatment of patients with PV resistant to or intolerant of hydroxyurea. Approval was based on data from the phase III RESPONSE trial, in which 222 patients with phlebotomy‐dependent PV and splenomegaly who had resistance or intolerance to hydroxyurea were randomised to ruxolitinib at a starting dose of 10 mg twice daily (n = 110) or best available therapy (n = 112).[69] At week 32 of the study, patients treated with ruxolitinib demonstrated superior haematocrit control and reductions in spleen volume compared with best available therapy (21% vs. 1%, respectively; p < 0.001). Ruxolitinib was generally well tolerated in the RESPONSE trial, with nearly 85% of ruxolitinib‐treated patients continuing to receive treatment after a median follow‐up of 81 weeks.[69] Other than ruxolitinib, pipobroman is approved in France as second‐line therapy following the failure of hydroxyurea, but is preferentially used in elderly patients due its leukemogenic potential.[61, 66]

In 2021, ropeginterferon alpha‐2b, a long‐acting, mono‐pegylated proline‐interferon alpha‐2b, became the first and only approved interferon for PV that patients can take regardless of their treatment history. The efficacy and safety of ropeginterferon alpha‐2b versus hydroxyurea were demonstrated in the phase III PROUD‐PV trial and its extension study, CONTINUATION‐PV, conducted in patients with early‐stage PV and no history of cytoreductive treatment or < 3 years of prior hydroxyurea treatment.[70, 71] At 36 months, ropeginterferon alpha‐2b resulted in a 71% haematologic response rate (defined as haematocrit < 45% with no phlebotomy in the past 3 months, platelet count < 400×10^9^/L and leucocyte count < 10×10^9^/L) compared to 51% for hydroxyurea (p = 0.012).[70, 71] The phase II low‐PV RCT has also recently shown that supplementing phlebotomy with ropeginterferon alpha‐2b is safe and effective in steadily maintaining haematocrit values < 45% in 127 low‐risk patients with PV.[72] Compared to phlebotomy and low‐dose aspirin (standard arm), the addition of ropeginterferon alpha‐2b was associated with a 84% haematological response rate (defined as haematocrit < 45% without disease progression) during a 12‐month period versus 60% in the standard arm (p = 0.0075). Overall, findings from the low‐PV study might pave the way for changing the current management of a subset of patients with low‐risk PV.[72]

The quality of life of patients with PV has been one of our group's main focuses during the last years. Management of PV‐associated symptoms, such as pruritus, erythromelalgia and migraine‐type headaches, are critical to improve patients’ quality of life.[63] Studies evaluating PV‐associated symptom control with hydroxyurea and/or phlebotomy demonstrate that these treatment modalities fare poorly. For instance, in a prospective evaluation of 1334 patients with PV who had characterised symptom burden (i.e. fatigue, early satiety, abdominal discomfort, inactivity, concentration problems, night sweats, pruritus, bone pain, fevers and weight loss), patients treated with phlebotomy and/or hydroxyurea continued to experience more severe symptoms compared with untreated patients.[73] In another prospective cohort study of 441 patients with PV, 301 (68.3%) experienced pruritus. In only 17 of these 301 patients (5.6%), PV‐directed therapy, including phlebotomy or cytoreduction, resolved pruritus. Only 72 patients (23.9%) received pruritus‐specific treatment, mostly antihistamines, which ameliorated symptoms in about half of them.[74] Overall, these data underscore the need for improved treatments to help alleviate common and problematic symptoms associated with PV. Of note, our group always offers sperm cryopreservation to male patients wishing to preserve their fertility before starting treatment for any MPN including PV and PMF.

### 6.1 Our group's practical applications

- Although therapeutic phlebotomy is the mainstay of controlling erythrocytosis, with a target haematocrit < 45%, it can exacerbate iron deficiency, which may cause thrombocytosis and additional symptoms. However, since the aim of therapeutic phlebotomy is to induce iron deficiency such that RBC production becomes limited, we usually do not prescribe iron supplementation.
- Our group usually reserves cytoreductive therapy (hydroxyurea or pegylated interferon alpha) for patients with high‐risk PV, and for low‐risk patients in the following situations:
- ○Presence of symptomatic or progressive splenomegaly.
- ○Persistent leucocytosis and/or thrombocytosis.
- ○Poor tolerance to phlebotomy.
- ○Uncontrolled PV‐associated symptoms.
- ○Pregnancy (with administration of pegylated interferon only).
- We prescribe ruxolitinib as second‐line therapy for PV patients intolerant of or resistant to hydroxyurea. A switch to pegylated interferon may also be an option for some patients.
- For the management of PV‐associated symptoms, we favour the use of ruxolitinib, antihistamines, aprepitant, antidepressants or interferon‐α to treat pruritus. To help minimise erythromelalgia and migraines, we offer aspirin.

## 7 DISCUSSION

PMF and PV are heterogenous *BCR‐ABL1*‐negative MPNs that adversely impact patients’ quality of life and survival. Although healthcare professionals should aim for an integrated, multimodal approach combining clinical, laboratory, morphological and genetic features to be applied for an accurate diagnosis, there is great variability in the way PMF and PV are diagnosed and risk‐stratified in routine clinical practice. For instance, in a retrospective analysis conducted at 34 private practices and primary care centres in Germany among 1476 patients with PV, the mutational status of *JAK2* was not investigated in 23% of patients. Likewise, serum erythropoietin levels had only been determined in 40% of patients.[75] In another retrospective analysis from the United States of patient records from community oncology practices, nearly one‐third of a total of 491 evaluated patients with myelofibrosis did not receive a risk categorisation at diagnosis.[76] Consistently, we found a similar pattern in our clinical practice observations in France.

There is also a multitude of therapeutic management issues affecting the care of patients with PMF and PV. In PV, current therapeutic decisions are based on predictive clinical variables, such as age and history of thrombosis, without considering the disease itself and its symptomatic burden. Other issues related to the therapeutic management of PV include impaired quality of life from frequent phlebotomy, ineffective reduction of PV‐associated symptoms and a high rate (25%) of intolerance or resistance to hydroxyurea front‐line therapy.[68] Pegylated interferon alpha is an attractive treatment, as it can alter the natural history of PV by targeting the mutant clone and preventing clonal evolution that underlies disease progression. It has also shown efficacy and safety in both low‐risk and high‐risk PV.[70, 72]

In PMF, the only potentially curative treatment is AHSCT, but it is associated with important morbidity and mortality, with no consensus regarding the optimal AHSCT protocol and the timing of transplant. Moreover, although the JAK inhibitor ruxolitinib has clearly enriched the therapeutic armamentarium for PMF, patients can develop ruxolitinib‐refractory disease requiring alternative therapeutic approaches.[40] Other critical questions in the management of patients with PMF include pre‐AHSCT management of marked splenomegaly and management of blast phase disease.[33]

Given the enhanced understanding of molecular genetics of PMF and PV, many emerging mechanistic‐based targeted therapeutic agents have been evaluated in the last years for both diseases. Givinostat, a histone‐deacetylase inhibitor that selectively targets JAK2V617F cell growth, seems to be a promising novel drug for PV.[77] Navitoclax (a novel anti‐apoptotic B‐cell leukaemia 2 [Bcl‐2] inhibitor), imetelstat (a telomerase inhibitor) and pelabresib (a BET inhibitor) might have disease‐modifying effects in patients with PMF.[78, 79, 80]

Overall, given the complexity of PMF and PV management and the heterogeneity of the two diseases highlighted in this critical review, we strongly believe that all patients with PMF and PV should be managed by multidisciplinary teams, with close collaboration between different healthcare professionals including biologists, haematologists, pathologists and oncologists.

## AUTHOR CONTRIBUTION

All authors contributed to the collection of information and evidence, wrote the original draft and reviewed and edited the final draft. Jean‐Jacques Kiladjian coordinated this work.

## CONFLICT OF INTERESTS

Shanti Amé participated in advisory boards for Bristol Myers Squibb and Novartis. Fiorenza Barraco declares no competing financial interests. Jean‐Christophe Ianotto received speaker fees from Novartis. Eric Jourdan participated in advisory boards for Bristol Myers Squibb, Novartis and AbbVie. Jérôme Rey participated in advisory boards for Bristol Myers Squibb, Novartis and AbbVie. Jean‐François Viallard received speaker fees from AOP Orphan, Novartis, Bristol Myers Squibb, AbbVie and Gilead. Mathieu Wémeau participated in advisory boards for AbbVie, AOP Orphan, Bristol Myers Squibb and Novartis, and received speaker fees from Novartis. Jean‐Jacques Kiladjian received consulting fees from AbbVie and Novartis, speaker fees from AOP Orphan, Novartis and Bristol Myers Squibb, and participated in advisory boards for Incyte.

## FUNDING INFORMATION

This project was funded by Novartis France.

## Data Availability

Data sharing is not applicable to this article as no new data were created or analysed.
